# Total iridoid glycoside extract of *Lamiophlomis rotata* (Benth) *Kudo* accelerates diabetic wound healing by the NRF2/COX2 axis

**DOI:** 10.1186/s13020-024-00921-1

**Published:** 2024-03-22

**Authors:** Xiaoyu Geng, Ying Wang, Huan Li, Liang Song, Chen Luo, Xiaojie Gu, Haixin Zhong, Huilin Chen, Xinzhu Chen, Jianwei Wang, Zheng Pan

**Affiliations:** 1 College of Traditional Chinese Medicine, Chongqing Medical University, Chongqing, China; 2 Chongqing Key Laboratory of Traditional Chinese Medicine for Prevention and Cure of Metabolic Diseases, College of Traditional Chinese Medicine, Chongqing Medical University, No.1, Yixueyuan Road, Chongqing, China

**Keywords:** Iridoid glycoside, *Lamiophlomis rotata*, NRF2/COX2 axis, Diabetic wound healing

## Abstract

**Background:**

*Lamiophlomis rotata* (Benth.) *Kudo* (*L. rotata*), the oral Traditional Tibetan herbal medicine, is adopted for treating knife and gun wounds for a long time. As previously demonstrated, total iridoid glycoside extract of *L. rotata* (IGLR) induced polarization of M2 macrophage to speed up wound healing. In diabetic wounds, high levels inflammatory and chemotactic factors are usually related to high reactive oxygen species (ROS) levels. As a ROS target gene, nuclear factor erythroid 2-related factor 2 (NRF2), influences the differentiation of monocytes to M1/M2 macrophages. Fortunately, iridoid glycosides are naturally occurring active compounds that can be used as the oxygen radical scavenger. Nevertheless, the influence of IGLR in diabetic wound healing and its associated mechanism is largely unclear.

**Materials and methods:**

With macrophages and dermal fibroblasts in vitro, as well as a thickness excision model of *db/db* mouse in vivo, the role of IGLR in diabetic wound healing and the probable mechanism of the action were investigated.

**Results:**

Our results showed that IGLR suppressed oxidative distress and inflammation partly through the NRF2/cyclooxygenase2 (COX2) signaling pathway in vitro*.* The intercellular communication between macrophages and dermal fibroblasts was investigated by the conditioned medium (CM) of IGLR treatment cells. The CM increased the transcription and translation of collagen I (COL1A1) and alpha smooth muscle actin (α-SMA) within fibroblasts. With diabetic wound mice, the data demonstrated IGLR activated the NRF2/KEAP1 signaling and the downstream targets of the pathway, inhibited COX2/PEG2 signaling and decreased the interaction inflammatory targets of the axis, like interleukin-1beta (IL-1β), interleukin 6 (IL-6), apoptosis-associated speck-like protein (ASC), cysteinyl aspartate specific proteinase1 (caspase1) and NOD-like receptor-containing protein 3 (NLRP3).In addition, the deposition of COL1A1, and the level of α-SMA, and Transforming growth factor-β1 (TGF-β1) obviously elevated, whereas that of pro-inflammatory factors reduced in the diabetic wound tissue with IGLR treatment.

**Conclusion:**

IGLR suppressed oxidative distress and inflammation mainly through NRF2/COX2 axis, thus promoting paracrine and accelerating wound healing in diabetes mice.

**Graphical Abstract:**

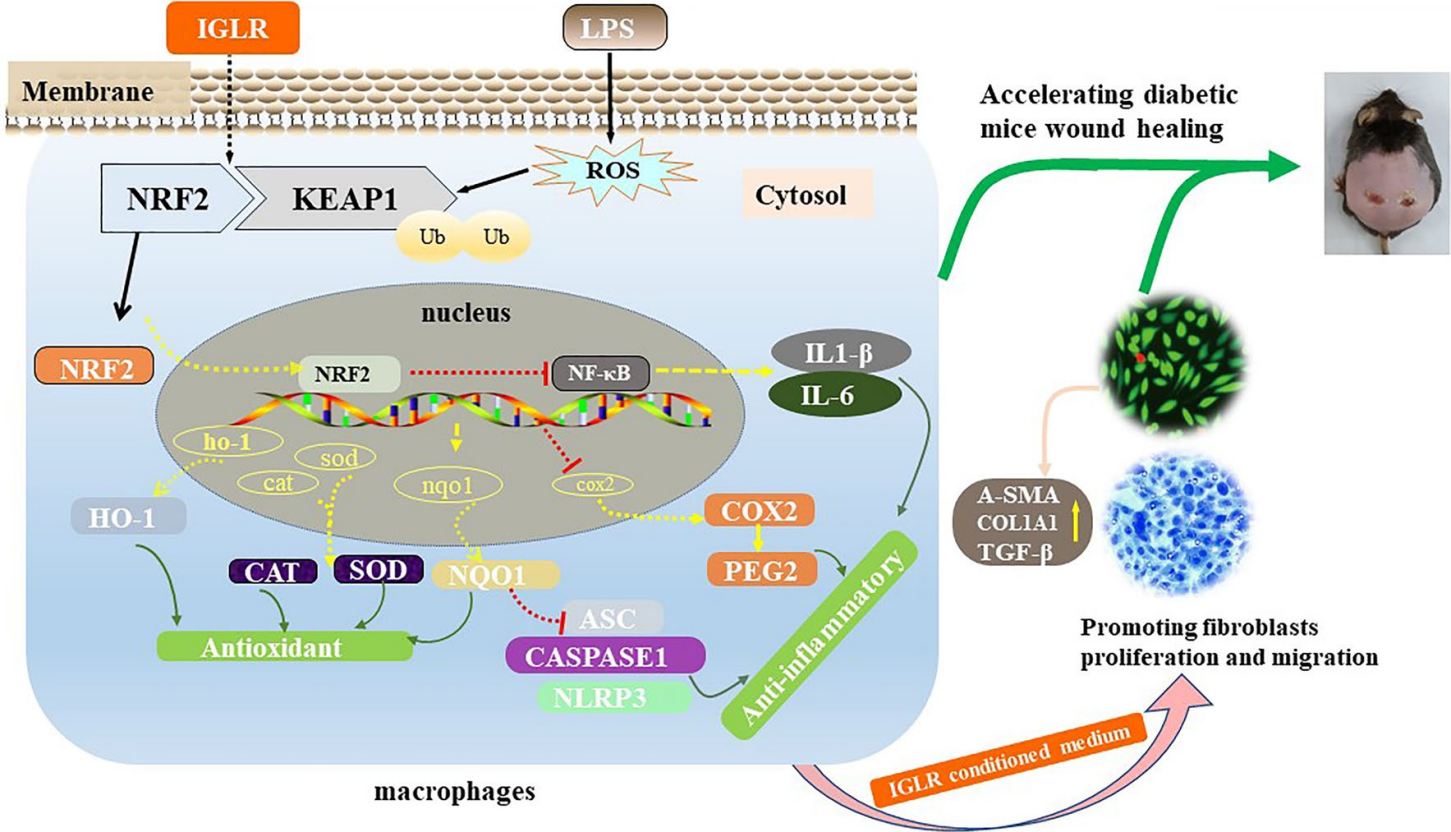

**Supplementary Information:**

The online version contains supplementary material available at 10.1186/s13020-024-00921-1.

## Introduction

Diabetes influences more than 422 million people globally, and impaired wound healing refers to a primary concern in patients suffering from diabetes [1]. In diabetic wounds, the healing is terminated during the inflammatory phase characterized by increased levels of reactive oxygen species (ROS), proteases, and proinflammatory cytokines, as well as cellular dysfunction [2, 3]. In addition, ischemia causes wound hypoxia, releasing more ROS, and increasing protein degradation [4]. Many small molecules that have been assessed as antioxidants show therapeutic potential in preclinical studies. However, the non-specific elimination of ROS using low-molecular-mass antioxidant complexes has not been a successful countermeasure against disease onset and progression in clinical trials over the past decades [5]. Fortunately, regulating ROS-targeted pathways through selective targeting, including nuclear factor erythroid 2-related factor 2 (NRF2) and nuclear factor-κB (NF-κB), provides a promising prospect for precise redox medicine in the future studies [6] continuous.

Wound healing refers to a complicated and highly orchestrated biological process, which usually involving hemostasis, inflammation, proliferation, and remodeling phases [7]. The inflammation phase is prolonged in diabetic wounds mainly owing to macrophage differentiation dysfunction, in which the M1 phenotype of macrophages dominates the microenvironment of the wound tissue, causing chronic inflammation and fibroblast senescence [8]. NRF2, a target gene of ROS, affects the differentiation of monocytes to M1/M2 macrophages [9]. NRF2-silencing mice exhibit hypersensitive to septic shock and continuous inflammation in wound healing [10], and the overexpression of NRF2 decreases inflammatory factors and chemokines expression in lipopolysaccharide (LPS)-induced macrophages [11, 12]. Furthermore, NRF2 regulates microglial dynamics, resulting in reduced production of nitric oxide synthase 2 (NOS2), cyclooxygenase2 (COX2), tumor necrosis factor (TNF), and IL-6, whereas increased expression of certain anti-inflammatory factors [13]. During the growth and repair processes, COX2, IL-1α, and NRF2 regulate the differentiated state of fibroblasts [14], which secrete various growth factors (GFs) during granulation tissue proliferation. Therefore, it is possible to investigate natural antioxidant compounds and extracts regulating the NRF2/COX2 axis for diabetic wound healing.

*Iridoid glycosides* (IGs) are observed in numerous medicinal plants with antioxidation, anti-inflammation, neuroprotective, and anti-tumor activities [15]. *Lamiophlomis rotata* (Benth.) *Kudo* (*L. rotata*), rich in iridoid glycosides, was used to treat traumatic injury in Tang dynasty. In previous studies, total iridoid glycoside extract of *L. rotata* (IGLR) showed wound healing effect [16]. Moreover, studies have confirmed that IGLR specifically reduces pain hypersensitivity by activating spinal glucagon-like peptide-1 receptors (GLP-1Rs) [17, 18]. Geniposide, a similar compound to iridoid glycoside in *L. rotata,* prevents oxidative stress, induces neuronal differentiation, and regulates insulin secretion by GLP-1Rs [19, 20]. A recent study revealed that 8‑*O*‑acetyl shanzhiside methylester, a quality control biomarker for *L. rotata*, showed neuroprotective effect on cognitive impairments induced by sleep deprivation as well as anxiety-like behaviors through modulating NRF2 and nuclear factor erythroid 2-related factor 2 (NRF2) pathways [21]. Loganin, another iridoid glycoside of *L. rotata*, ameliorates podocyte apoptosis in diabetic nephropathy though targeting advanced glycation end products (AGEs) and its receptor (RAGE) signaling [22], which play the role of as vital mediators in some downstream signaling cascades influencing immune-inflammatory responses and oxidative stress [23]. This evidence demonstrates that IGLR may provide a perspective on redox medicine for treatment of diabetic wounds through regulating inflammatory responses and oxidative stress. Nevertheless, its associated is still unknown.

In this study, using macrophages and dermal fibroblasts in vitro, as well as an overall skin wound model of genetically diabetic (*db/db*) mice in vivo, we explored antioxidant and anti-inflammatory impacts of IGLR on the communication between macrophages and dermal fibroblasts via the NRF2/COX2 axis and the potential use of IGLR in diabetic wound healing.

## Material and methods

### Materials

IGLR obtained in *L. rotata* at our laboratory was assayed through ultra high-performance liquid chromatography coupled with time-of-flight mass spectrometry (UPLC-QTOF-MS), which can be observed in our previous studies [24]. Briefly, we extracted the herbal material (1.9 kg) thrice using refluxing 70% EtOH, with removal of solvent at the decreased pressure, while the remaining solution was taken up via resin. The 35% ethanol elution of microporous adsorption resin was removed at reduced pressure, to obtain IGLR in the resultant extract (187.6 g). After dissolution into (0.1 g/mL), the sample was filtered with the 0.22-µm nylon membrane filter prior to UPLC analyses.

Preparation of aqueous extract of the herb (Duyiwei capsules, Batch No. 2001022301.) was provided by Duyiwei BioPharmaceutical Co., Ltd., (Gansu, China). High-glucose Dulbecco’s modified Eagle’s medium (DMEM) and Fetal Bovine Serum (FBS) were offered by Gibco (North America). Lipopolysaccharide (LPS) offered by Aladdin (Shanghai, China). All the remaining chemicals were analytically pure and used as purchased.

### ***UPLC-MS/MS***^***n***^*** analysis***

In line with the previous description, UPLC-MS/MS^n^ analysis was conducted [25]. Mobile phases system was different between two studies. In the present study, mobile phases included (a) water containing 0.1% (v/v) formic acid as well as (b) methanol containing 0.1% (v/v) formic acid. The elution procedure was shown below, 2-min holding at 5% B, 5–15 min at the gradient of 5%–15%, 12–22 min at 15%–25% B, 22–34 min at 25%–35% B, and then back to 5% B in 1 min. The flow rate, column temperature and injection volume were 0.25 mL/min, 35 °C and 2 µL, separately.

### Antioxidant assay

The DPPH and ABST^+^ assays were used for free radical scavenging potential of IGLR according to a previously published protocol [26], with ascorbic acid being the standard. The absorbance values were detected at 517 and 734 nm, respectively. We determined radical scavenging activity (%) as follows,

$${\text{Percentage}}\;{\text{effect}} = {\text{Abs}}_{{{\text{control}}}} {-}{\text{Abs}}_{{{\text{sample}}}} /{\text{Abs}}_{{{\text{control}}}} \times {1}00.$$

### Cell culture conditions and CM preparation

RAW 264.7 mouse macrophages, and L929 dermal fibroblasts were provided by Fu Heng Bio (Shanghai, China), and cultivated within DMEM that contained 10% FBS, and 1% penicillin streptomycin under 37 °C and 5% CO_2_ conditions. IGLR at 0, 50, 100, and 200 μg/mL was added into 0.1% DMSO. Additionally, we cultivated and RAW 264.7 cells (1 × 10^6^/well) within 6-well plates, followed by 4-h pre-treatment using LPS (1 μg/mL), and then 48-h co-cultured with DMSO or IGLR. The supernatants were collected as the IGLR conditioned medium (IGLR-CM) for the communication of fibroblasts and macrophages [27].

### The proliferation and migration of fibroblasts by IGLR-CM

Live and dead cell counts with Calcein-AM / propidium iodide (PI) [28] staining kit (Beyotime Biotechnology Co., Ltd, China). In brief, the L929 cells incubated by IGLR-CM were collected and rinsed by PBS. Then, cells were co-incubation using 1 μM AM and 9 μM PI for a 15-min period within the cell incubator. After washing, the fluorescence microscope (THUNDER Imager system, Leica, Germany) was used to observe the cells.

In Transwell assay, we suspended 1 × 10^4^ cells/well into the low serum (5% FBS) before seeding in upper Transwell chamber (Corning, Corning, NY, USA; pore size, 8 μm), with 3 replicates being set in each group. Next, 10% FBS-containing complete medium IGLR-CM was introduced into bottom chamber for the period of 12 h. Cells adherent onto upper membrane surface were removed, while migrating cells onto lower surface were subjected to 0.5% crystal violet staining (Beyotime Biotechnology Co., Ltd, China). Cell migration was observed using optical microscope.

### Animal treatment

Experiments on animals were performed in line with laboratory animal use and care guidelines from Animal Ethics Committee of Chongqing Medical University. The 8-week-old adult male *db/db* (C57BLKS/J-leprdb/leprdb) mice (40–45 g) and age-matched heterozygous control *db/m* mice (20–23 g) were offered by Hunan SJA Laboratory Animal Co., Ltd., (Hunan, China), raised under constant conditions (25 ± 2 °C, 40–60% humidity, 12-h/12-h light/dark cycle), with free access to standard feed and water.

Animals were acclimatized and fed for a week in the animal center to establish wound model with 8 mm in diameter as described in literature [29]. Then, *db/m* mice were divided as positive controlled group, *db/db* mice were randomized as 4 groups: vehicle group, Duyiwei capsules group (DYW-C group, 0.8 g/kg per day, equal to 4.0 g/kg of raw drug), L-IGLR group (0.1 g/kg per day, equal to 1.0 g/kg of raw drug), H-IGLR group (0.4 g/kg per day, equal to 4.0 g/kg of raw drug). Drug was supplemented within 0.3% Sodium carboxymethylcellulose (CMC-Na) dilution. In each group, animals were given gavage of 0.3% CMC-Na at an equal amount. There were 12 animals per group, with each individual mouse fed in a separate cage, and receiving daily gavage treatment for 14 consecutive days. Subsequently, the wounds were observed and isometric photographs were taken on days 0, 3, 7, 10 and 14. A rectangular ruler was used to record the wounds. Wound area was calculated with the use of Image J 1.49p software, and the final wound healing rate (%) was analyzed: Wound healing rate (%) = (A_0_ − A_n_)/A_n_ × 100% (A_0_ and An Wound area on Day 1 and n, respectively, cm^2^).

In this case, half of the 12 mice in each group were sacrificed on day 7 and 14 after surgery. After anesthesia through i.p. injection of sodium pentobarbital, we obtained blood and skin samples and preserved them under − 80 °C before the following analysis. Later, partial skin tissues were immersed in 10% neutral-buffered formalin to conduct pathology analyses.

### Real-time PCR analysis

We isolated total RNA in day 7/14 skin wound tissue and IGLR-treated RAW264.7 cells (1 × 10^6^ /well within the 6-well plate) through the collection of specimens. After rinsing with phosphate buffered saline (PBS), the TRIzol reagent (Takara, Shiga, Japan) was used for extraction following the specific instructions. cDNA was prepared through reverse transcription using Reverse Transcription Kit (AG, Hunan, China). With SYBR® Premix TaqTM II (Tli RNaseH Plus) (Takara, Shiga, Japan), we conducted qRT-PCR. Additional file 1: Table S1 displays primer sequences. Gene expression was explored with the use of the 2-ΔΔCt method [30].

### Western blot analysis

In line with the previous results, Western Blot was performed [31]. Briefly, total proteins from cells or skin trauma tissues were isolated with RIPA lysis buffer that contained proteinase/phosphatase inhibitor (Beyotime Biotechnology Co., Ltd, China). Then, BCA protein assay kit (Beyotime Biotechnology Co., Ltd, China) was utilized to analyze total protein content. Later, 10% SDS-PAGE gels were applied in separating protrin aliquots before transfer on polyvinylidene fluoride (PVDF; Millipore, USA) membranes. After blocking using milk for 2-h,overnight incubation was performed under 4 °C using primary antibodies, and later using horseradish per-oxidase-labeled secondary antibodies (antibody information is shown in Additional file 1: Table S2). ECL reagents were applied to visualize chemiluminescence signals (Advansta, CA, USA). ImageJ software was used for blot quantification.

### Histological and immunohistochemical evaluation

Traumatic wound tissue from mice including the center of the lesion was subjected to fixation using 4% paraformaldehyde overnight, ethanol dehydration, and transparentizing using xylene, prior to paraffin embedding. The 5-μm thin tissue sections were made to conduct hematoxylin and eosin (H&E) staining to visualize tissue injury and inflammation, while collagen deposition during healing was observed with Masson trichrome staining in line with the previous description [32]. In this study, we applied immunofluorescent double-staining in labeling antioxidant stress factors Nrf2 and HO-1, and inflammatory factors IL-1β and IL-6, collagen fibril generation markers α-SMA, COL1A1 and TGF-β, respectively. Fluorescence in situ hybridization (FISH) was performed with the SweAMI probe-FISH + IF protocol from servicebio technology (Wuhai, China), which was used in combination with immunofluorescence (IF) and FISH methods for COX2 and PGE2 levels in paraffin-embedded skin sections based on previous methods [33].

### ELISA

For the purpose of measuring MDA, SOD, and CAT contents, we used biochemical assay kits (Shanghai Enzyme-linked Biotechnology Co., Ltd. Shanghai, China). Biochemical assay kits (Jiangsu Jingmei Biotechnology Co., Ltd. China) were adopted for detecting levels of PGE2, IL-6, vascular endothelial growth factor (VEGF) and epidermal growth factor (EGF). Following the specific-introduction manual, all assays were carried out.

### Statistical analysis

Data were indicated by mean ± SD from 3 or more separate assays, and examined with two-tailed student t-test, and linear regression or one way variance analysis with Tukey’s post-hoc test. Statistical analysis was completed with GraphPad Prism software version 8.0 (GraphPad Software Inc., USA). *P* ≤ 0.05 stood for statistical significance.

## Results

### Chemical composition in IGLR through UPLC-Q/TOF/MS^n^

**Fig. 1:**
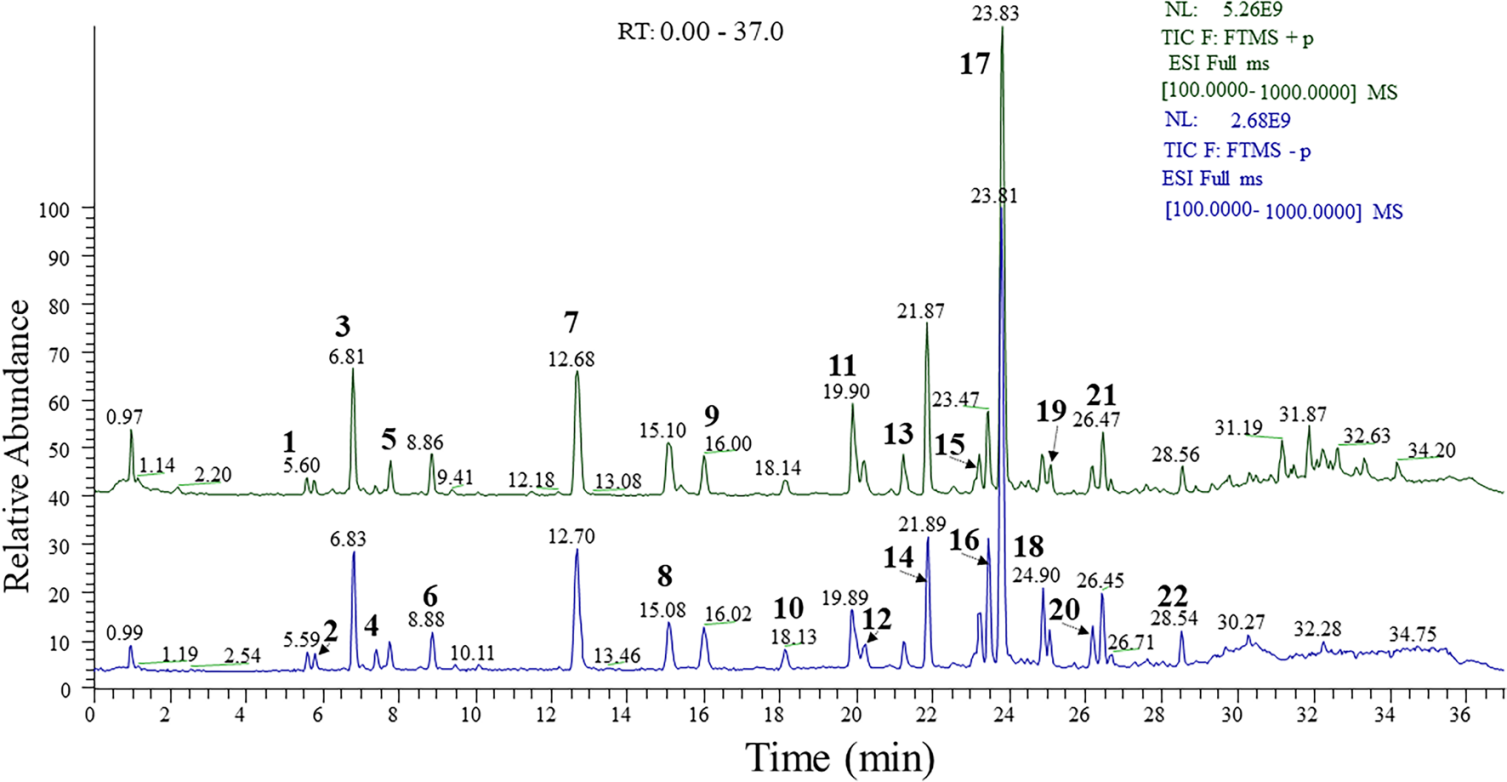
Total ion current chromatograms showing components of iridoid glycoside extract of *L. rotata* with the positive (green) and negative (blue) ion modes

MS and MS^n^ were used for analyzing total IGLR in positive/negative ion modes (Fig. 1). All molecular formulas were calculated by the ions of [M+Na]^+^ or [M+H]^+^ under positive ion mode, as well as that of [M−H]^−^ under negative ion mode. Data were verified with characteristic ions of iridoid glycoside, which has been reported in previous studies on *L. rotata* and *Lamium* specie in the databases PubChem and SciFinder. Main compounds in IGLR, including seventeen iridoid glycoside, and five phenolic glycosides (Table 1), were obtained based on retention time and mass dates for the above compounds in previous studies [25].

**Table 1:** The main compounds identified in IGLR by UPLC-Q/TOF-MS^n^

NO	RT (min)	Compound name	Formula	Calculated (Da)	Selected ion	Error (ppm)	Selected ion	Error (ppm)
1	5.60	7-Epi-Phlomiol	C_17_H_26_O_13_	438.1373	[M+Na]^+^	− 1.39	[M−H]^−^	1.62
2	5.81	Schismoside	C_17_H_26_O_12_	422.1424	[M+Na]^+^	− 0.90	[M−H]^−^	1.21
3	6.81	Phlorigidoside C	C_17_H_24_O_11_	404.1319	[M+Na]^+^	− 1.64	[M−H]^−^	2.97
4	7.41	Decaffeoylacteoside	C_20_H_30_O_12_	462.1737	[M+Na]^+^	− 0.66	[M−H]^−^	1.80
5	7.77	Lamalbide	C_17_H_26_O_12_	422.1424	[M+Na]^+^	− 1.17	[M−H]^−^	1.28
6	8.86	Penstemoside	C_17_H_26_O_11_	406.1475	[M+Na]^+^	− 1.07	[M−H]^−^	2.20
7	12.66	Shanzhiside methyl ester	C_17_H_26_O_11_	406.1475	[M+Na]^+^	− 0.86	[M−H]^−^	0.64
8	15.12	6-*O*-Acetylshanzhi-side methyl ester	C_19_H_28_O_12_	448.1581	[M+Na]^+^	− 0.93	[M−H]^−^	1.12
9	16.02	Phloyoside II	C_17_H_25_ClO_12_	456.1034	[M+Na]^+^	− 0.90	[M−H]^−^	2.00
10	18.12	Chlorotuberroside	C_17_H_25_ClO_11_	440.1085	[M+Na]^+^	− 1.06	[M−H]^−^	1.43
11	19.92	7,8-Dehydropenste-monoside	C_17_H_20_O_10_	388.1369	[M+Na]^+^	− 1.61	[M−H]^−^	1.35
12	20.18	7,8-Dehydropenste-moside	C_17_H_24_O_11_	404.1319	[M+Na]^+^	− 1.64	[M−H]^−^	1.27
13	20.92	8-*O*-Acetylshanzhi- side	C_18_H_26_O_12_	434.1424	[M+Na]^+^	− 1.56	[M−H]^−^	1.56
14	21.84	Loganin	C_17_H_26_O_10_	390.1526	[M+Na]^+^	− 2.23	[M−H]^−^	1.17
15	23.22	Deoxyloganic acid	C_16_H_24_O_9_	360.14204	[M+Na]^+^	− 2.32	[M−H]^−^	2.00
16	23.46	Forsythoside B	C_34_H_44_O_19_	756.24769	[M+Na]^+^	− 0.04	[M−H]^−^	1.39
17	23.82	8-*O*-Acetylshanzhi-side methyl ester	C_19_H_28_O_12_	448.1581	[M+Na]^+^	− 1.38	[M−H]^−^	1.72
18	24.85	Verbascoside	C_29_H_36_O_15_	624.20543	[M+Na]^+^	− 0.68	[M−H]^−^	1.33
19	25.10	Alyssonoside	C_35_H_46_O_19_	770.26334	[M+Na]^+^	− 0.23	[M−H]^−^	1.57
20	26.18	7-*O*-benzoylloganic-acid	C_21_H_36_O_12_	480.16317	[M+Na]^+^	− 1.79	[M−H]^−^	2.63
21	26.43	Lamiophlomio-side A	C_35_H_46_O_21_	802.25316	[M+H]^+^	− 3.81	[M−H]^−^	1.12
22	28.54	6-*O*-syringylbarlerin	C_28_H_36_O_16_	628.20034	[M+Na]^+^	− 2.13	[M−H]^−^	1.18

### Antioxidant activity of IGLR in vitro

**Fig. 2:**
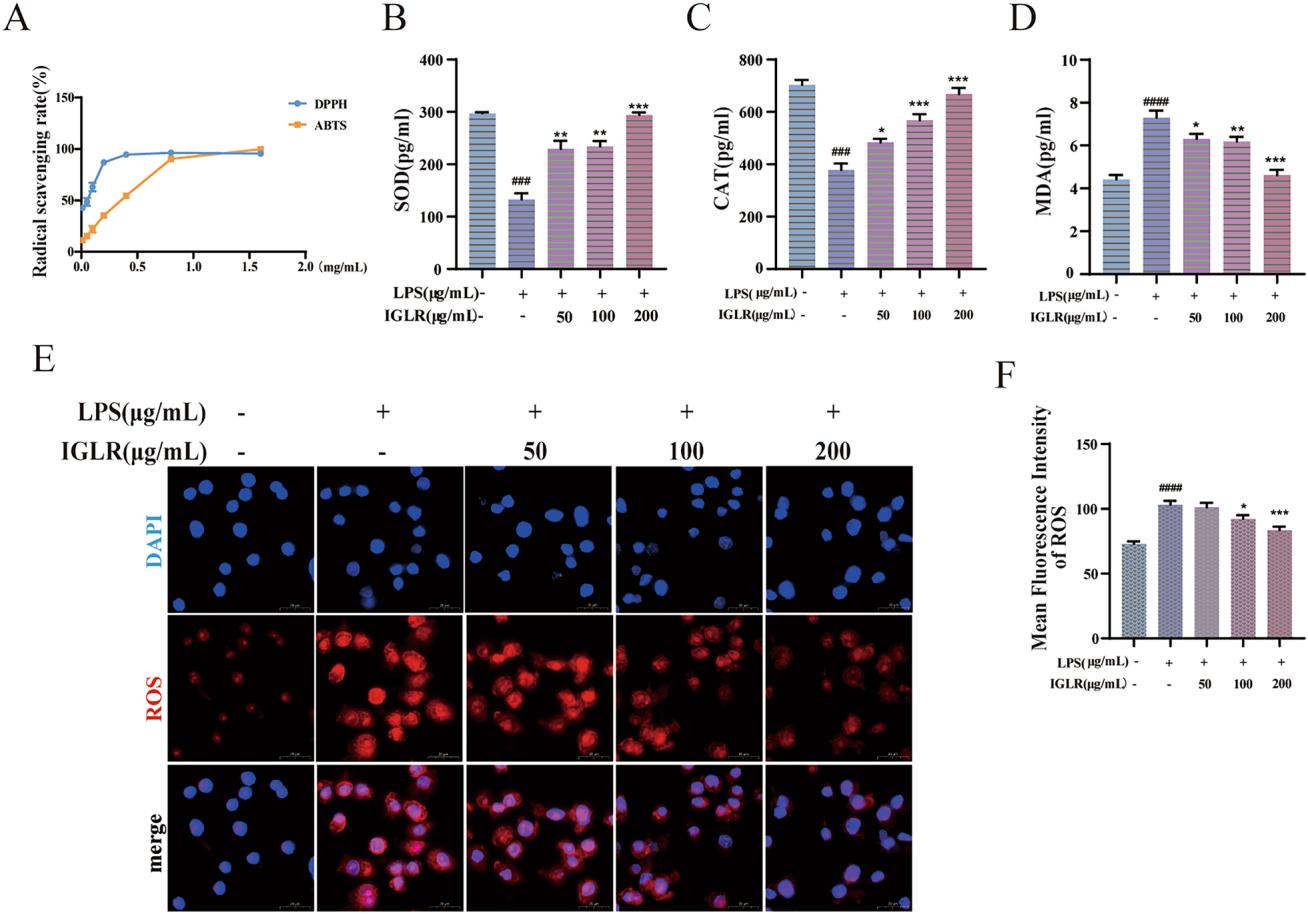
IGLR scavenges oxygen free radicals. **A** The scavenging rate of ABTS^+^ and DPPH by IGLR. **B**–**D** Roles of IGLR (0, 50, 100 and 200 μg/mL) in SOD, CAT and MDA levels in macrophages by LPS (1 μg/mL) induced for 48 h. **E** IGLR effects on ROS in macrophages cells under 48-h LPS (1 μg/mL) stimulation. **F** Quantification of NRF2 and HO-1 during immunofluorescence analysis (n = 3). ^*###*^*P* < 0.005,^*####*^*P* < 0.001 versus positive control group, *P < 0.05,**P < 0.01, ***P < 0.005 versus LPS (1 μg/mL) stimulated group, one-way ANOVA and Tukey’s multiple comparison test. In addition, results are shown to be means ± SEM

In diabetic wounds, several pathologic mechanisms lead to ROS accumulation, causing wound healing complications. The antioxidant activity of IGLR was explored by ABTS^+^ and DPPH radical scavenging assays. As shown in Fig. 2A, IGLR inhibited ABTS^+^ and DPPH free radicals, exhibiting IC_50_ values of 120 ± 5.3 and 420 ± 12.6 μg/mL, respectively. To demonstrate the antioxidant effects of the extract, this study examined the MDA, SOD, and CAT contents in LPS-stimulated macrophages with or without IGLR treatment. ELISA assays suggested that IGLR significantly accelerated the release of SOD and CAT and inhibited that of MDA (Fig. 2B–D). A similar trend as MDA was observed for ROS production with the use of IF following IGLR treatment (Fig. 2E, F). Collectively, our results confirm that IGLR exhibits antioxidant activity by lowering the intracellular generation of ROS and increasing the secretion of SOD and CAT in a dose-dependent manner.

### IGLR suppresses oxidative distress and inflammation via the NRF2/COX2 signaling pathway

#### IGLR suppresses oxidative distress via NRF2/KEAP1 signaling pathway

**Fig. 3:**
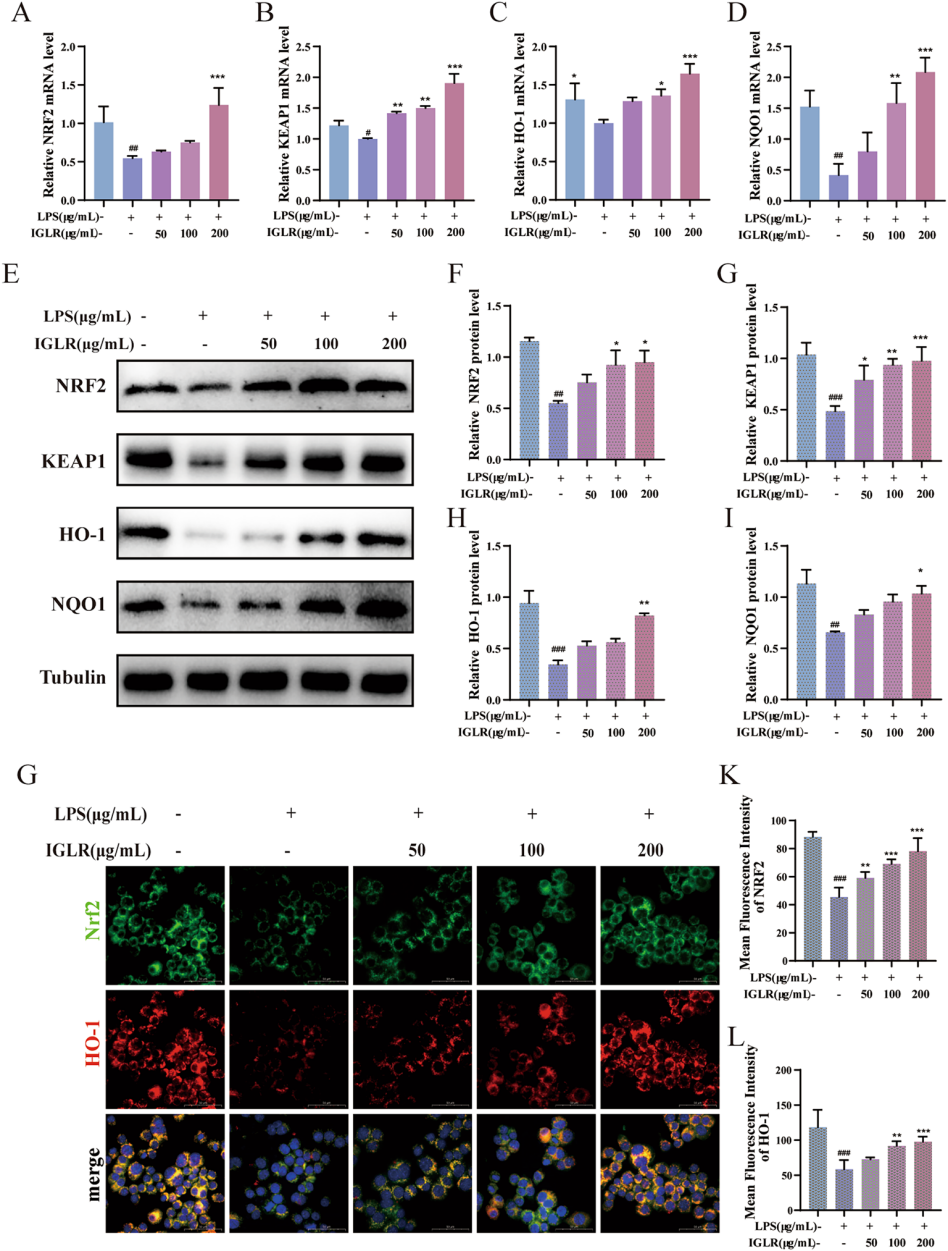
IGLR stimulates macrophage resistance to oxidative stress via NRF2/KEAP1 pathway. **A**–**D** NRF2, KEAP1, HO-1 and NQO1 mRNA levels by real-time PCR in LPS stimulated macrophages with IGLR treatment. **E** The western blots of NRF2, KEAP1, HO-1 and NQO1in LPS induced macrophage with 48-h IGLR (0, 50, 100 and 200 μg/ mL) treatment. **F**–**I** Quantitative results of (E) (n = 3). **J** Immunofluorescence analysis on HO-1 (red) and NRF2 (green) within macrophages with different concentrations of IGLR (scale bar, 50 μm). **K**, **L** Quantification of NRF2 and HO-1 levels during immunofluorescence analysis (n = 3). ^*#*^*P* < 0.05, ^*##*^*P* < 0.01, ^*###*^*P* < 0.005 versus positive control group, *P < 0.05, **P < 0.01, ***P < 0.005 versus LPS (1 μg/mL) group, one-way ANOVA and Tukey’s multiple comparison test. In addition, results are shown to be means ± SEM

We further explored the possible mechanisms of IGLR in suppressing oxidative distress. As shown in Fig. 3, IGLR dosage-dependently promoted NRF2, kelch-like each-association protein (KEAP1), heme oxygenase-1 (HO-1), and NAD(P)H: quinone oxidoreductase 1 (NQO1) transcription (Fig. 3A–D). Western blotting analysis indicated that different concentrations of IGLR up-regulated levels of NRF2, KEAP1, HO-1 and NQO1 proteins (Fig. 3E–I). To illuminate whether IGLR accelerated NRF2 and downstream proteins, IF was labeled with NRF2 and HO-1; therefore, positive NRF2 and HO-1 expression in 100 and 200 μg/mL IGLR groups significantly increased relative to control group (Fig. 3G–L). Therefore, IGLR suppresses oxidative distress via the NRF2/KEAP1 signaling pathway.

#### IGLR suppresses inflammation by the COX2/PEG2 signaling pathway

**Fig. 4:**
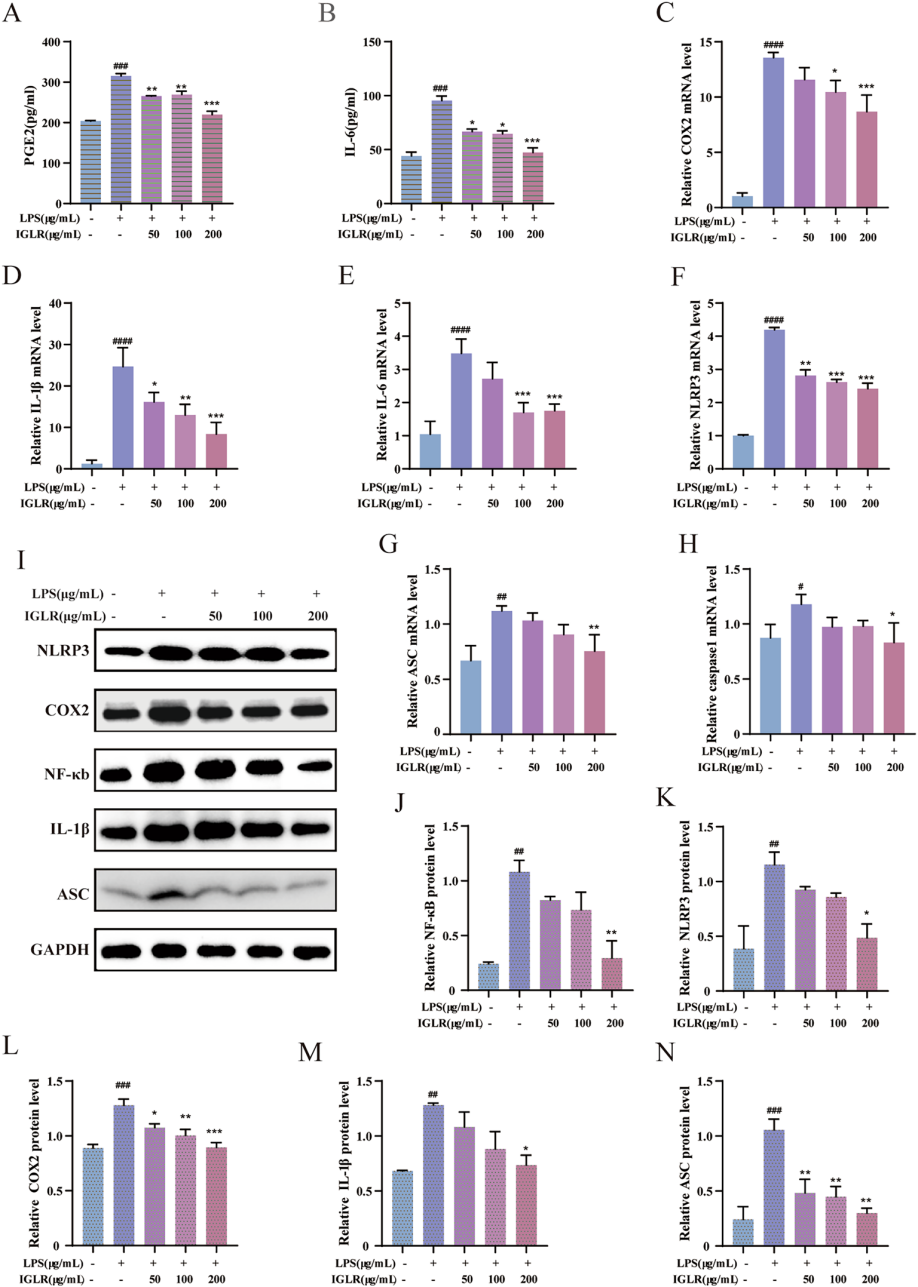
IGLR enhances macrophage anti-inflammation through COX2/PGE2 signaling pathway. **A**, **B** Functions of IGLR (0, 50, 100 and 200 μg/mL) in PGE2 and IL-6 levels in LPS induced macrophages for 48 h. **C**–**H** COX2, IL-1β, IL-6, NLRP3, ASC and caspase1 mRNA expression with real-time PCR in LPS induced macrophages. **I** The western blots of COX2, NLRP3, NF-κB, IL-1β, and ASC within LPS could induce macrophages under 48-h IGLR (0, 50, 100 and 200 μg/ mL) treatment. **J**–**N** Quantitative results for (I) (n = 3). ^*#*^*P* < 0.05, ^*##*^*P* < 0.01, ^*###*^*P* < 0.005, ^*####*^*P* < 0.001 versus control group, *P < 0.05, **P < 0.01, ***P < 0.005 versus LPS (1 μg/mL) group, one-way ANOVA and Tukey’s multiple comparison test. Results are shown to be means ± SEM

A recent study reported NRF2 was a vital regulatory factor for immunomodulatory genes, which suppresses oxidative stress to induce COX2, and is essential for the secretion of prostaglandin E2 (PEG2) and is strongly triggered by H_2_O_2_ or TNFα only in the existence of NRF2 [34]. ELISA indicated that IGLR treatment of LPS-stimulated cells significantly reduced PEG2 and IL-6 production (Fig. 4A, B). RT-PCR revealed that IGLR down-regulated inflammatory factors COX2, ASC, caspase 1, and NLRP3, and chemotactic factors of IL-1β and IL-6 (Fig. 4C–H). Similarly, western blotting showed that COX2, NLRP3, NF-κB, IL-1β, and ACS levels in macrophages were significantly suppressed after treatment with IGLR (Fig. 4I–N). collectively, IGLR exerts antioxidant and anti-inflammatory effects through the NRF2/COX2 signaling axis.

#### IGLR suppresses activation of oxidative distress to induce fibroblasts via a paracrine mechanism

**Fig. 5:**
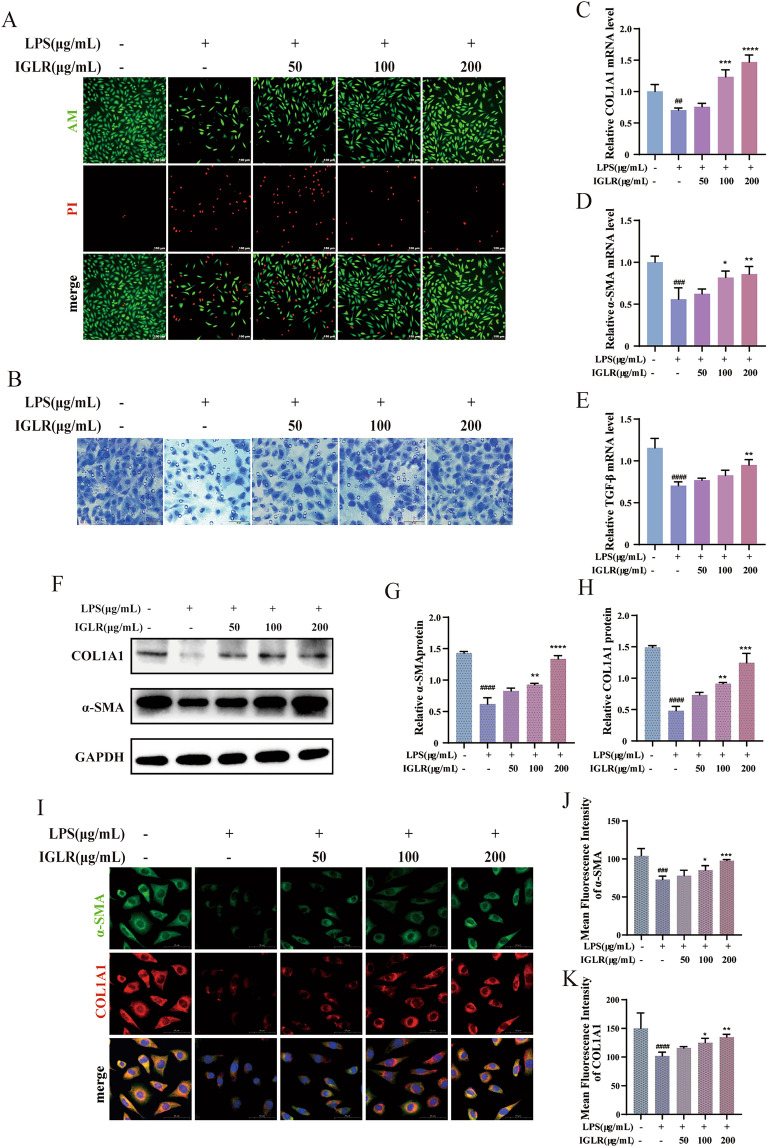
IGLR conditioned medium (IGLR-CM) improves the proliferation and migration of fibroblasts. **A** Calcein (green) and PI (red) staining for proliferation in L929 cells by 24-h IGLR-CM incubation at diverse concentrations (scale bar, 100 μm). **B** Cell transfer after 24 h incubation of fibroblasts with different concentrations of IGLR-CM (scale bar, 50 μm). **C**–**E** α-SMA, TGF-β and COL1A1 mRNA expression analyzed via real-time PCR in fibroblasts at 24 h of IGLR-CM intervention. **F** COL1A1 and α-SMA protein expression within L929 cells incubated with different concentrations of IGLR-CM. **G**, **H** Quantification of western blots (n = 3). **I** Immunofluorescence analysis on COL1A1 (red) and α-SMA (green) within L929 cells with IGLR-CM (scale bar, 50 μm). **J**, **K** Quantification of COL1A1 and α-SMA during immunofluorescence analysis (n = 3). ^*##*^*P* < 0.01, ^*###*^*P* < 0.005, ^*####*^*P* < 0.001 versus control group, *P < 0.05, **P < 0.01, ***P < 0.005 versus LPS (1 μg/mL) group, one-way ANOVA and Tukey’s multiple comparison test. Results are shown to be means ± SEM

To explore the effect of IGLR in regulating fibroblast and macrophage communication in the microenvironment of wounds, IGLR-CM was prepared and cultured with L929 cells (dermal fibroblasts). The combination of calcein-AM/PI staining indicated that IGLR-CM significantly promoted fibroblast proliferation (Fig. 5A). The results of the Transwell assay further suggested that IGLR-CM accelerated fibroblast migration in a dosage-dependent manner (Fig. 5B). In fibroblasts, IGLR-CM significantly up-regulated COL1A1, α-SMA, and TGF-β mRNA expression (Fig. 5C–E) after incubation for 24 h. Western blotting (Fig. 5F–H) and IF (Fig. 5I–K) showed that IGLR-CM obviously elevated the expression of α-SMA (a myofibroblast phenotype marker) and COL1A1 (produces collagen I to be the main structural protein for ectodermal matrix for wound repair) [8, 35]. These data suggest that IGLR promotes fibroblast activation and collagen production to facilitate diabetic wound healing based on a paracrine mechanism.

#### IGLR significantly enhances wound healing within a holistic skin wound model of genetically diabetic (db/db) mice

**Fig. 6:**
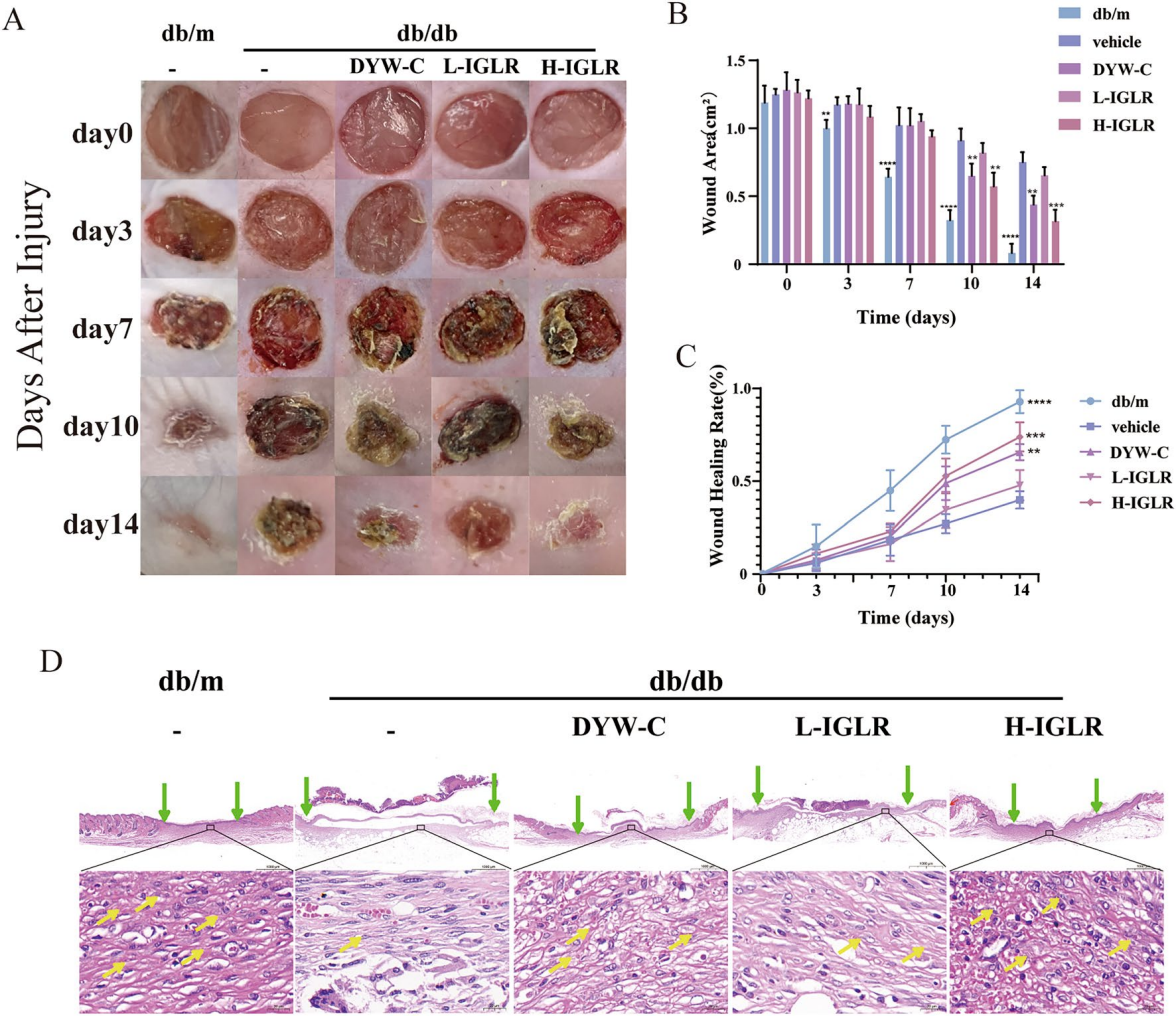
IGLR significantly promotes wound healing in *db/db* mice. **A** Typical images showing wound area within diabetic mice during 0 ~ 14 days. Quantitative analysis of wound area (**B**) and wound closure rates (**C**) at 0, 3, 7, 10 and 14 days following IGLR treatment at different concentrations (n = 5). **D** Wound skin tissue was subjected to HE staining 14 days postoperatively (scale bar, 1000 μm, 50 μm), green arrows indicate wound extent with or without IGLR treatment, the yellow arrows show granulation tissue

In order to explore the function of IGLR in diabetic repair in wounds, the normality of the wound extent and granulation tissue was measured in *db/m* mice, which were assigned to the positive control group. Moreover, commodity preparation using the aqueous extract of the herb (Duyiwei capsules; DYW-C) was applied as the herbal control group. Wound healing rates were measured on days 0, 3, 7, 10, and 14 in the five groups. IGLR exhibited a weaker wound closure effect than the positive control group; however, it visibly narrowed the wound area compared with the vehicle group (Fig. 6A, B), and H-IGLR (400 mg/kg) group revealed a faster wound closure rate than that of the same dose of DYW-C group, especially on day 14 (Fig. 6C). On the 14th day after surgery, the pathomorphology of the cutaneous wounds was assessed using H&E staining; the H-IGLR (400 mg/kg) group showed a thicker dermis and more granular tissue in the wound than those observed in the DYW-C and vehicle groups (Fig. 6D), further demonstrating the pharmacological effects of IGLR on promoting diabetic wound healing. Surprisingly, IGLR made no obvious function in VEGF and EGF levels within wound tissues (Additional file 1: Figure S1). As dozens of growth factors are secreted by macrophages, fibroblasts, keratinocytes, and endothelial cells in the microenvironment of wound tissue [36], further studies need to be investigated which growth factors are involved in IGLR-induced diabetic wound repair.

### IGLR promotes diabetic wound healing via the NRF2/COX2 signaling pathway

#### IGLR promotes diabetic wound via the NRF2/KEAP1 signaling pathway

**Fig. 7:**
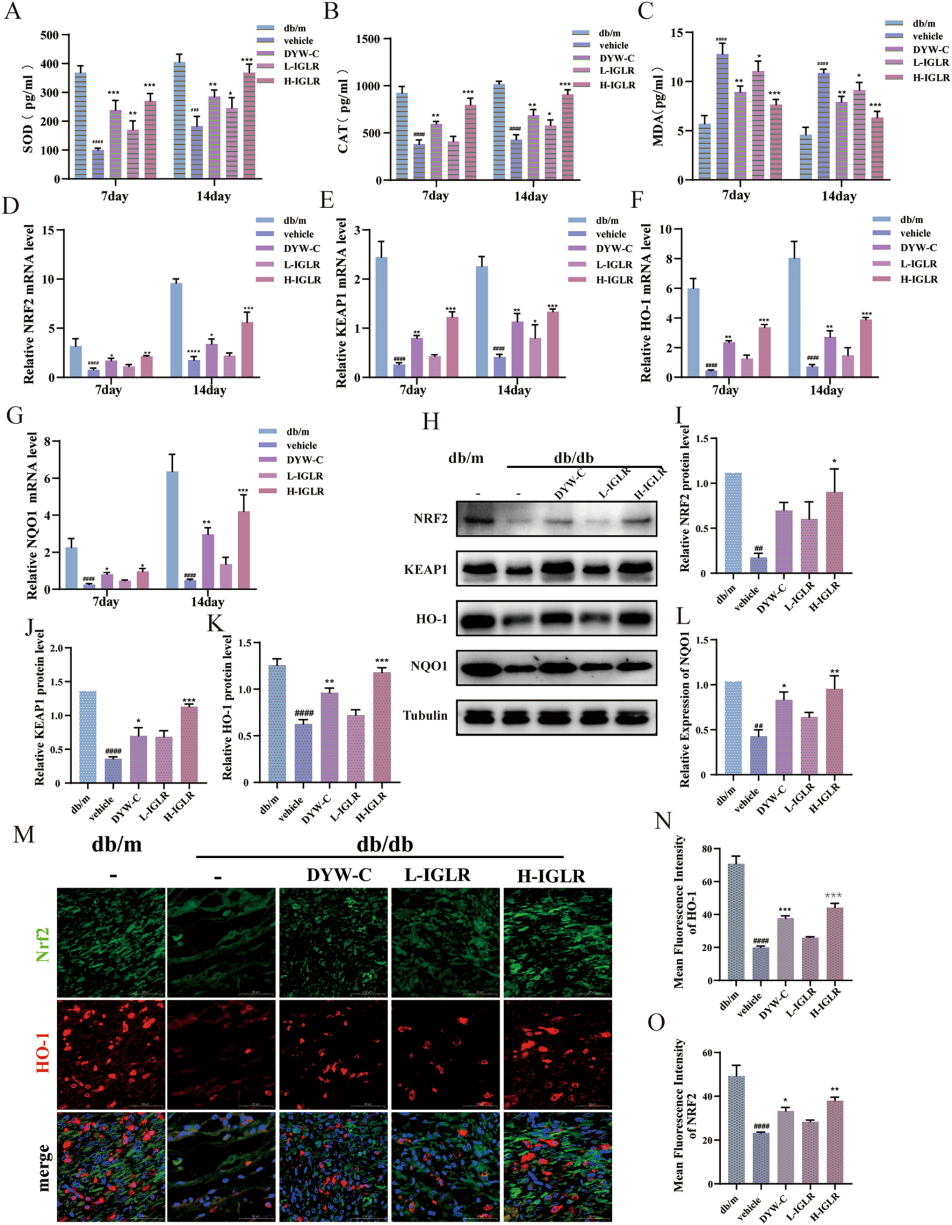
IGLR accelerates diabetic wound repair via NRF2/Keap1 pathway. **A**–**C** SOD, CAT, and MDA levels within plasma after gavage of IGLR at varying concentrations for 7 and 14 days (n = 6). **D**–**G** The NRF2, KEAP1, HO-1 and NQO1 mRNA levels within wound tissues detected by qRT-PCR 7 and 14 days later (n = 6). **H** NRF2, KEAP1, HO-1 and NQO1 expression in wound tissue of diabetic mice was detected by Western blot at Days 14 with or without IGLR treatment. **I**–**L** The quantitative results of (H) (n = 3). (**M**) Immunofluorescence analysis on HO-1 (red) and NRF2 (green) within wound tissues of diverse groups (scale bar, 50 μm). **N**, **O** Quantification of NRF2 and HO-1 in immunofluorescence staining (n = 3). ^*##*^*P* < 0.01, ^*####*^*P* < 0.001 versus positive control group, *P < 0.05, **P < 0.01, ***P < 0.005 versus vehicle group, one-way ANOVA and Tukey’s multiple comparison test. Results are shown to be means ± SEM

To determine the observed mechanisms in vitro, we performed an in vivo study*.* At First, the impacts of IGLR on SOD, CAT, and MAD in a diabetic wound mouse model after 7 and 14 days were determined using ELISA. IGLR could notably elevate the levels of SOD (Fig. 7A) and CAT (Fig. 7B) but decreased the level of MDA (Fig. 7C), regardless of whether the drug was administered for 7 or 14 days. RT-PCR results suggested that IGLR up-regulated NRF2, KEAP1, HO-1, and NQO1 mRNA expression in mice skin tissue on days 7 and 14, with better up-regulation of anti-oxidative stress genes observed on day 14 post-surgery (Fig. 7D–G). In addition, we investigated NRF2, KEAP1, HO-1, and NQO1 protein expression in diabetic wound tissue at day 14 by Western blotting. As expected, the H-IGLR (400 mg/kg) group significantly upwardly adjusted NRF2, KEAP1, HO-1, and NQO1 protein levels (Fig. 7H–L). Double immunofluorescence staining confirmed NRF2 and HO-1 levels in tissue in diabetic mice treated with IGLR (Fig. 7M–O). These findings demonstrate that IGLR can accelerate wound healing in diabetic mice activating the NRF2/KEAP1 pathway.

#### IGLR promotes diabetic wound through the COX2/PEG2 signaling pathway

**Fig. 8:**
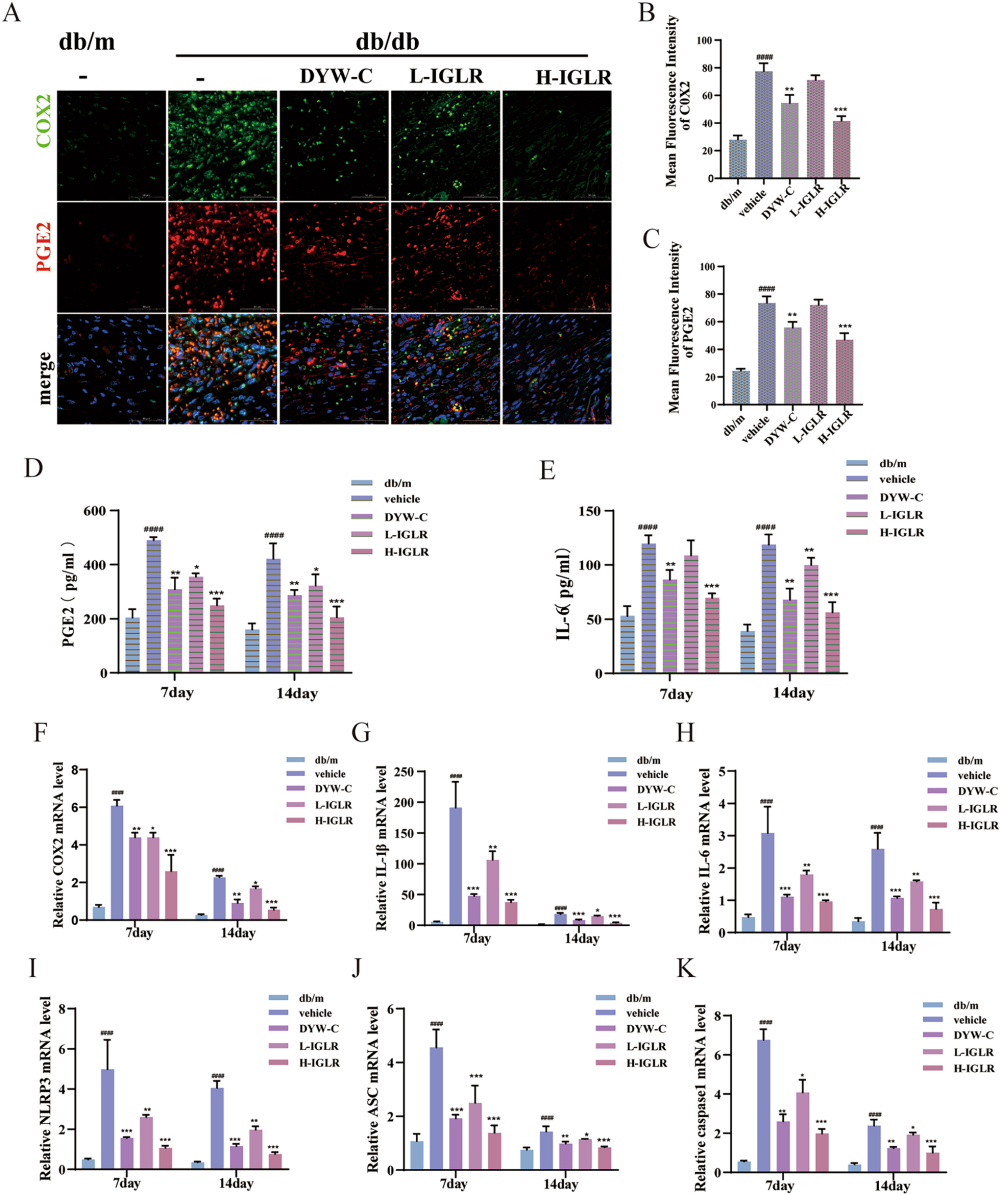
IGLR facilitates diabetic wound closure through the COX2/PEG2 signaling pathway. **A** IF and FISH double staining for COX2 (green) and PGE2 (red) in wound tissue at Day 14 post surgery (scale bar, 50 μm). **B**, **C** Quantification of COX2 and PGE2 through IF and FISH analyses (n = 3). **D**, **E** PGE2 and IL-6 levels in plasma of diabetic mice at day 7 and 14 of gavage of different doses of IGLR; **F**–**K** COX2, IL-1β, IL-6, NLRP3, ASC and caspase1 mRNA expression within wound tissues detected by qRT-PCR after 7 and 14 days (n = 5). ^*####*^*P* < 0.001 versus positive control group, *P < 0.05, **P < 0.01, ***P < 0.005 versus vehicle group, one-way ANOVA and Tukey’s multiple comparison test. Results are shown to be means ± SEM

**Fig. 9:**
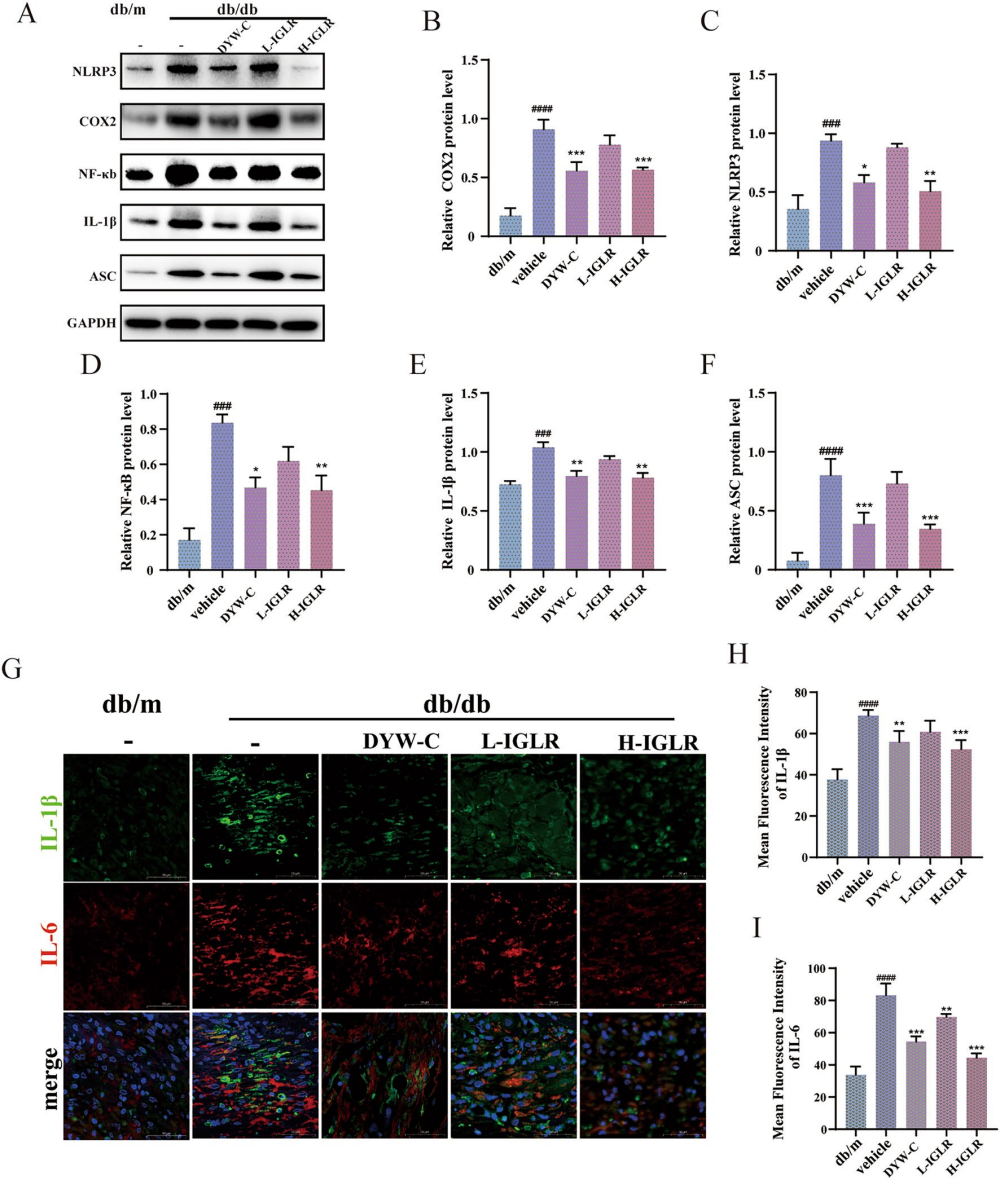
IGLR promotes diabetic wound closure through the COX2/PEG2 signaling pathway. **A** The Western blot assay showed COX2, NLRP3, NF-κB, IL-1β, and ASC levels in wound tissues of genetically modified mice after 14 days of IGLR treatment. **B**–**F** Quantification of western blots of (**A**) (n = 3). **G** Immunofluorescence analysis of IL-6 (red) and IL-1β (green) within wound tissues at 14 days postoperatively (scale bar, 50 μm). **H**, **I** Quantification of IL-6 and IL-1β in immunofluorescence staining (n = 3). ^*###*^*P* < 0.005, ^*####*^*P* < 0.001 versus positive control group, *P < 0.05, **P < 0.01, ***P < 0.005 versus vehicle group, one-way ANOVA and Tukey’s multiple comparison test. Results are shown to be means ± SEM

IF combined with RNA FISH was used for assaying proteins and RNA molecules involved in wound tissue inflammation. Relative to control group, COX2 and PGE2 activities in skin wounds of the IGLR-treated group were lower, as evidenced by IF and FISH examination (Fig. 8A–C). The plasma levels of PGE2 and IL-6 were measured, and IGLR obviously lowered the levels of these inflammatory factors (Fig. 8D, E). In parallel, we detected the expression of inflammation-related genes via RT-PCR. The findings demonstrated COX2, IL-1β, IL-6, NLRP3, ASC, and caspase1 genes levels of H-IGLR group visibly depressed compared with that of the model group (Fig. 8F–K). As expected, Western blotting indicated that the COX2, NLRP3, NF-κB, IL-1β, and ACS expression was up-regulated for model group, and significantly down-regulated for H-IGLR group (Fig. 9A–F). These results were verified by IF, showing that IGLR decreased IL-1β and IL-6 expression in wound tissue of diabetic mice (Fig. 9G–I). In conclusion, these findings indicate that IGLR promotes wound healing by enhancing antioxidant stress and attenuating inflammatory responses in diabetic mice largely via the NRF2/COX2 signaling axis.

#### IGLR promotes diabetic wound healing via a paracrine mechanism

**Fig. 10:**
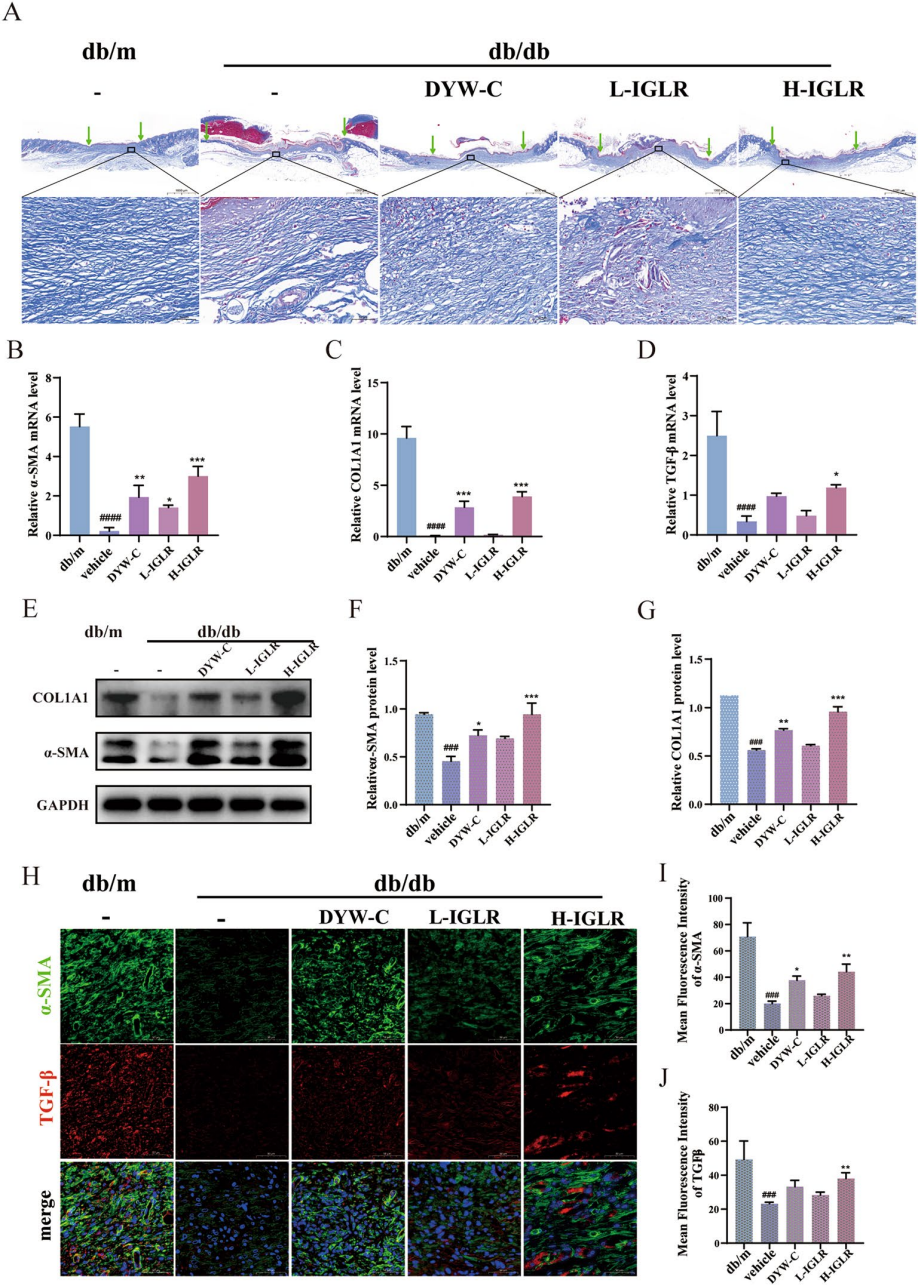
IGLR contributes to the wound healing of diabetic wounds through a paracrine mechanism. **A** Masson trichrome staining for wound skin tissues 14 days postoperatively in diabetic mice (scale bar, 1000 μm, 50 μm). **B**–**D** α-SMA, TGF-β and COL1A1 mRNA levels within wound tissues in diabetic mice detected through qRT-PCR 14 days later (n = 6). **E** The effect of α-SMA and COL1A1 levels on collagen production in the *db/db* mice wound model after 14 days of drug administration detected by Western blot assay. **F**, **G** The quantitative results of **E**. **H** Immunofluorescence analysis on TGF-β (red) and α-SMA (green) within wound tissues in diabetic mice after 14-day IGLR treatment at varying doses (scale bar, 50 μm). **I**, **J** Quantification of TGF-β and α-SMA during immunofluorescence analysis (n = 3), ^*###*^*P* < 0.005, ^*####*^*P* < 0.001 versus positive control group, *P < 0.05, **P < 0.01, ***P < 0.005 versus vehicle group, one-way ANOVA and Tukey’s multiple comparison test. Results are shown to be means ± SEM

In vitro*,* we demonstrated that IGLR suppressed oxidative distress and inflammation through the NRF2/COX2 axis. This signaling pathway makes a vital impact on the translation of M1 macrophages into the M2 phenotype, which secretes various growth factors and promotes the deposition of the extracellular matrix (ECM) in diabetic wound healing [37]. To investigate whether IGLR accelerates the closure of chronic wounds via the NRF2/COX2 axis through crosstalk between fibroblasts and macrophages, on day 14, Masson’s trichrome staining, Western blotting, and IF were performed on the wound tissues. As indicated by Masson’s trichrome staining, IGLR-treated wound tissue was more mature and had more collagen deposition than control group (Fig. 10A). Moreover, α-SMA, collagen Ι, and TGF-β mRNA levels significantly increased with IGLR (400 mg/kg) treatment in diabetic mice (Fig. 10B–D). Similarly, Western blotting showed that wounds treated with IGLR exhibited significantly up-regulated α-SMA and COL1A1 protein levels (Fig. 10E–G). In addition, we further examined TGF-β and α-SMA contents by IF, that IGLR obviously enhanced their positive areas within diabetic trauma tissue (Fig. 10H–J). Based on the obtained results, IGLR notably enhances collagen deposition and promotes granulation tissue formation in diabetic mice, possibly by stimulating fibroblasts through a paracrine mechanism.

## Discussion

Diabetes mellitus is a metabolic disorder in which patients experience hyperglycemia inducing oxidative stress in the tissue microenvironment [5]. Type 2 diabetes is recognized a “redox disease” and the various roles of oxidants in regulating insulin signaling are currently well recognized [38]. The NRF2/KEAP1 axis is a physiological thiol-based sensor-effector apparatus which responds to oxidative challenges and acts in maintaining eukaryotic redox homeostasis. Increased awareness of NRF2 as the possible drug target has stimulated interest in pharmaceutics and resulted in significant investment in developing NRF2 modulators [39]. Resveratrol, sulforaphane, and triterpenoids are agonists of NRF2 [40, 41]. Iridoid glycosides are naturally occurring active compounds, such as oxygen radical scavengers. *L. rotata* is rich in iridoid glycosides, including 8-*O*-acetylshanzhiside methyl ester or shanzhiside methyl ester, which are labeled compounds used for quality control of herbal medicines and related preparations [24]. In a recent study, 8-*O*-acetylshanzhiside methyl ester demonstrated its anti-anxiety effect through the NRF2 signaling pathway [21]. Our previous research has shown that IGLR is responsible for the wound-healing effect of *L. rotata*. The current work investigated the impacts of IGLR on promoting the healing of diabetic wounds via the NRF2/COX2 signaling pathway.

NRF2 is a low-abundance protein with a short half-life of around 15–40 min according to cell type. Significantly, KEAP1 comprises some high-activity cysteine residues that, once modified by electrophilic molecules, stops it from targeting NRF2 for proteasomal degradation [42]. In this study, IGLR significantly up-regulated NRF2 and KEAP mRNA and protein levels in LPS-mediated macrophages. The obtained findings indicate that IGLR facilitates the stability of NRF2 by up-regulating KEAP1 in vitro. In addition, the NRF2 transcriptional targets, HO-1 and NQO1, were assayed. The results suggested that IGLR activated the transcription and translation of NRF2-target genes, which was further confirmed by NRF2 and HO-1 double staining. This evidence indicates that IGLR is a potential redox agent that promotes transcription and translation, which can prevent NRF2 degradation.

The NRF2 and NLRP3 inflammasomes physically interact with each other to regulate inflammatory responses [21], and the NRF2/KEAP1 pathway makes a vital impact on the regulation of inflammation through protein–protein interactions with NF-κB, COX2, and NLRP3 [43]. To explore the possible anti-inflammatory mechanisms following NRF2/KEAP1 pathway activation via IGLR, PEG2 and IL-6 levels were assayed by ELISA in LPS-induced macrophages treated with IGLR, and then COX2, NLRP3, ACS, IL-1β, and NF-κB protein and mRNA expression was determined. These findings demonstrated that IGLR lowered the generation of inflammatory factors and decreased the release of chemokines through the COX2/PEG2 signaling pathway. Therefore, IGLR inhibits inflammation and oxidative distress partially through NRF2/COX2 pathway.

Wound healing is a complicated but highly coordinated biological process, which is primarily related to macrophages, fibroblasts, keratinocytes, and endothelial cells [44]. A higher exposure of H_2_O_2_ than the physiological intracellular level (≤ 100 nM) results in an inflammatory response, cell death and growth arrest, through a variety of mechanisms [5]. In this study, IGLR significantly raised plasma levels of SOD and CAT, while lowering the production of ROS and MDA. It has been indicated that chronic wounds increase oxidative stress through increasing MDA enzyme activity and decreasing SOD and CAT levels via the Nrf2 pathway [45, 46]. Herein, IGLR suppressed oxidative distress in the wound model of mice. According to IF analysis, NRF2 and HO-1 levels in diabetic tissues showed a positive relationship to IGLR treatment. FISH and ELISA assays revealed that IGLR negatively regulated the transcription and translation of COX2 and PEG2, along with the interaction of inflammatory signaling targets, like IL-1β, IL-6, NLRP3, ASC, and caspase1. Activation of the COX2/PEG2 pathway suppresses natural killer cell function, causes chronic inflammation, and regulates the epithelial-mesenchymal transition of wound tissue [47, 48]. Therefore, our results show that the NRF2/COX2 signaling pathway is vital for diabetic wound healing after IGLR treatment.

Most fibroblasts in chronic wounds senesce prematurely, exhibit abnormal morphology, and how a reduced ability to migrate and proliferate [49, 50]. Oxidative stress exerts a vital impact on the regulation of diabetic wound healing through the development and maturation of the ECM [51]. To investigate the interaction between macrophages and fibroblasts after treatment with IGLR, CM from IGLR-treated macrophages was collected and used to culture fibroblasts [16]. Therefore, IGLR-CM facilitated fibroblast activation, proliferation, and migration; increased SOD and CAT release outside the cells; and reduced MDA release (Additional file 1: Figure S2). As a result, IGLR-CM led to negative feedback to oxidative stress in fibroblasts interacting with macrophages, which was further confirmed by NRF2, KEAP1, NQO1, and HO-1 mRNA levels within fibroblasts as measured by RT-PCR (Additional file 1: Figure S3). CM increased the transcription and translation of α-SMA and collagen I, which are markers of the activated and restored functionality of fibroblasts, respectively [52, 53]. Collectively, IGLR may promote cell communication and accelerate wound closure by suppressing oxidative distress and inflammation via the NRF2/COX2 axis. Masson’s trichrome staining revealed a favorable action of IGLR on collagen deposition in wound tissue. Additionally, qPCR and Western blotting showed that IGLR dramatically enhanced α-SMA and collagen I mRNA and protein levels. This was further verified by IF showing that the TGF-β and α-SMA positive area significantly increased on the wound skin of diabetic mice after IGLR treatment. Consequently, IGLR suppresses oxidative distress and inflammation mainly through the NRF2/COX2 axis, thereby promoting paracrine signaling and accelerating wound healing in diabetic mice.

Studies have shown that iridoid glycosides are agonists of GLP-1R; however, unexpectedly, IGLR shows no major changes in either HbA1c or body weight (Additional file 1: Figure S4), conforming to the results of studies on diabetic wound healing with dipeptidyl peptidase 4 inhibitors. They can prolong the half-life of endogenous GLP-1 and are classic antihyperglycemic agents used across the world in managing type 2 diabetes [54]. This indicates that the role of IGLR in improving repair of diabetic wounds is unrelated to glycemic control, and further studies need to be performed to investigated other mechanisms [28].

## Conclusion

*L. rotata* has been used to treat knife and gun wounds for centuries in Traditional Tibetan Medicine. According to the obtained findings, total iridoid glycoside extract of *L. rotata* suppressed oxidative distress and inflammation through the NRF2/COX2 axis, thereby promoting paracrine signaling and accelerating wound healing in diabetic mice.

### Supplementary Information

**Additional file 1: Table S1.** The primer sequences used in qRT-PCR experiments. **Table S2.** The antibodies used for the experiment. **Figure S1.** IGLR had no obvious effect on VEGF and EGF in the wound tissue. **Figure S2.** IGLR-CM scavenges oxygen free radicals. **Figure S3.** IGLR-CM remarkably elevated the level of antioxidative stress factors in fibroblasts. **Figure S4.** IGLR not significantly altered body weight and HbA1c levels in *db/db* mice.

## Data Availability

Data can be obtained from corresponding author on request.

## Abbreviations

ASC: Apoptosis-associated speck-like protein
α-SMA: α-Smooth muscle actin
CAT: Catalase
CMC-Na: Sodium carboxymethylcellulose
COX2: Cyclooxygenase2
caspase: Cysteinyl aspartate specific proteinase
COL1A1: Collagen I
DMEM: Dulbecco’s modified Eagle’s medium
EGF: Epidermal Growth Factor
FISH: Fluorescence in situ hybridization
HbA1C: Glycosylated hemoglobin
H&E: Hematoxylin and eosin
IF: Immunofluorescence
HO-1: Heme Oxygenase-1
IL-1β: Interleukin-1β
IL-6: Interleukin 6
IGLR: Total iridoid glycoside extract of *Lamiophlomis rotate*
KEAP1: Kelch-like each-association protein
LPS: Lipopolysaccharide
*L. rotata*: *Lamiophlomis rotata* (Benth)* Kudo*
Masson: Masson’s trichrome staining
MDA: Malondialdehyde
NF-кB: Transcription factor nuclear factor-kappa B
NLRP3: NOD-like receptor-containing protein 3
NRF2: Nuclear Factor erythroid 2-Related Factor 2
NQO1: NAD(P)H:quinone oxidoreductase 1
PBS: Phosphate Buffered Saline
PEG2: Prostaglandin E2
PMSF: Phenylmethanesulfonyl fluoride
PVDF: Polyvinylidene fluoride membranes
ROS: Reactive oxygen species
RT-qPCR: Real-time Quantitative Polymerase Chain Reaction
SOD: Superoxide dismutase
TGF-β: Transforming growth factor β
UPLC-Q/TOF/MSn: Ultrahigh performance liquid chromatography coupled with time-of-flight mass spectrometry
VEGF: Vascular endothelial growth factor
