# Supplementary material for: Total iridoid glycoside extract of Lamiophlomis rotata (Benth) Kudo accelerates diabetic wound healing by the NRF2/COX2 axis
Source: Chin Med. 2024 Mar 22;19:53. doi: 10.1186/s13020-024-00921-1 (PMC10960394; doi:10.1186/s13020-024-00921-1)
Supplement: Supplementary file 1 — Additional file 1: Table S1. The primer sequences used in qRT-PCR experiments. Table S2. The antibodies used for the experiment. Figure S1. IGLR had no obvious effect on VEGF and EGF in the wound tissue. Figure S2. IGLR-CM scavenges oxygen free radicals. Figure S3. IGLR-CM remarkably elevated the level of antioxidative stress factors in fibroblasts. Figure S4. IGLR not significantly altered body weight and HbA1c levels in db/db mice. [file 13020_2024_921_MOESM1_ESM.docx]

**Additional Material**

**Table S1**. The primer sequences used in qRT-PCR experiments

| **Name** | **Species** | **Forward Primer** | **Reverse Primer** |
| --- | --- | --- | --- |
| GAPDH | mouse | TGTTTCCTCGTCCCGTAGA | ATCTCCACTTTGCCACTGC |
| Nrf2 | mouse | GCACATCCAGACAGACACCAG | TATCCAGGGCAAGCGACTCAT |
| HO-1 | mouse | ACTCCCTGTGTTTCCTTTCTCT | CTGGATGTGCTTTTGGTGAGG |
| Keap1 | mouse | TTGTCTCCCCACATCTTCTTTGA | AACACTAAGCGAACACCAAGC |
| NQO1 | mouse | CAGCCAATCAGCGTTCGGTA | CTTCATGGCGTAGTTGAATGATGTC |
| COX2 | mouse | GCTCCTTTTCAACCAGCAGTT | GGGGTGCCAGTGATAGAGTG |
| IL-1β | mouse | TTCCCCAGGGCATGTTAAGG | GTCTTGGCCGAGGACTAAGG |
| IL-6 | mouse | CTTCTTGGGACTGATGCTGGT | GGTCTGTTGGGAGTGGTATCC |
| NLRP3 | mouse | CCTCTTTGGCCTTGTAAACCAG | TGGCTTTCACTTCAATCCACT |
| ASC | mouse | GAAGTGGACGGAGTGCTGGAT | TCATCTTGTCTTGGCTGGTGGT |
| Caspase1 | mouse | ACTCGTACACGTCTTGCCCTCA | CTGGGCAGGCAGCAAATTC |
| VEGF | mouse | GACCGATTAACCATGTCACC | CCAAAGTGCTCCTCGAAG |
| EGF | mouse | TGATAGCCAGCTCCAATCTACTG | CCAGTCCTCTTGTTCACCCTTAT |
| Col1A1 | mouse | CAGAGGCGAAGGCAACA | GTCCAAGGGAGCCACATC |
| α-SMA | mouse | GCCCAGAGCAAGAGAGG | TGTCAGCAGTGTCGGATG |
| TGF-β | mouse | CCATTGCTGTCCCGTGCAGA | CGAAAGCCCTGTATTCCGTCT |

**Table S2.** The antibodies used for the experiment

| **Antibodies** | **Manufacturer** | **Catalog number** |
| --- | --- | --- |
| NRF2 (D1Z9C) XP^®^ Rabbit mAb | CST | #12721 |
| Cox2 (D5H5) XP^®^ Rabbit mAb | CST | #12282 |
| GAPDH(D16H11) XP^®^ Rabbit mAb | CST | #5174 |
| NF-κB p65(D14E12) XP^®^ Rabbit mAb | CST | #8242 |
| α-Smooth Muscle Actin(D4K9N) XP^®^ | CST | #67648 |
| COL1A1(E8F4L) XP^®^ Rabbit mAb | CST | #72026 |
| HO-1/HMOX1 Polyclonal antibody | Proteintech | 10701-1-AP |
| KEAP1 Polyclonal antibody | Proteintech | 10503-2-AP |
| IL-1 Beta Polyclonal antibody | Proteintech | 26048-1-AP |
| NQO1 Polyclonal antibody | Proteintech | 11451-1-AP |
| Anti-NLRP3 antibody [EPR23094-1] | Abcam | ab263899 |
| Anti-TMS1/ASC antibody [EPR23978-28] | Abcam | ab283684 |
| TGF beta 1 Antibody | Affinity | AF1027 |

| **A** | **B** | **C** | **D** |
| --- | --- | --- | --- |
| 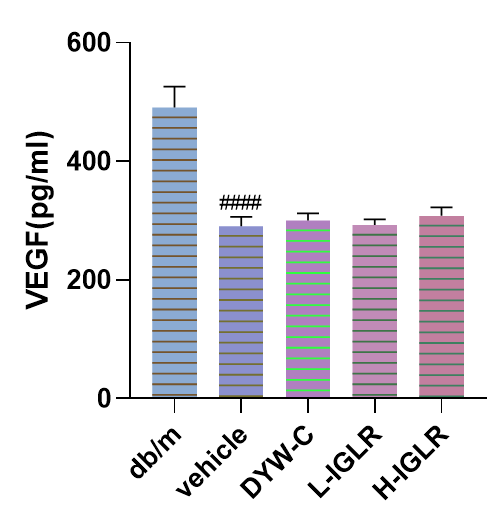 | 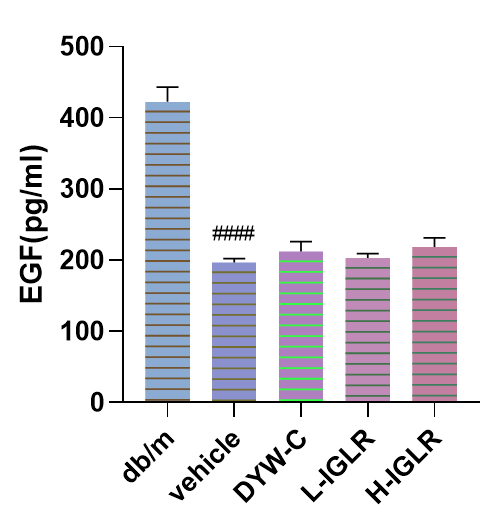 | 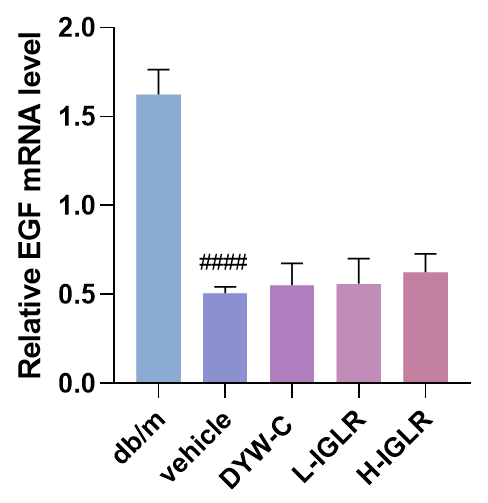 | 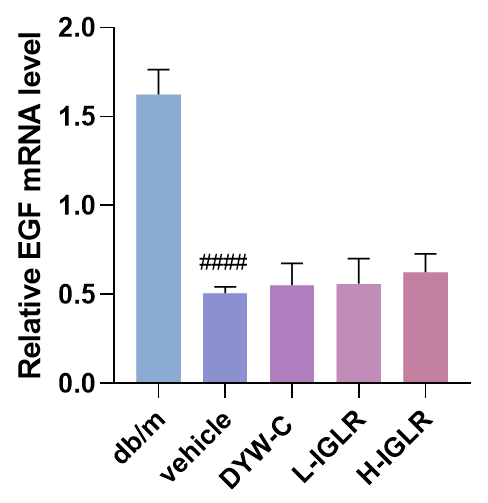 |

**Figure S1.** IGLR had no obvious effect on VEGF and EGF in the wound tissue. (**A, B**) Effect of IGLR on VEGF and EGF in wound tissue on day 14 by ELISAs with IGLR treatment (n = 6); (**C, D**) The mRNA levels of VEGF and EGF on in wound tissue on day 14 with IGLR treatment (n = 5); *^###^P* < 0.005 compared with control group, one-way ANOVA with Tukey’s multiple comparison test. All data are reported as the means ± SEM.

| **A** | **B** | **C** |
| --- | --- | --- |
| 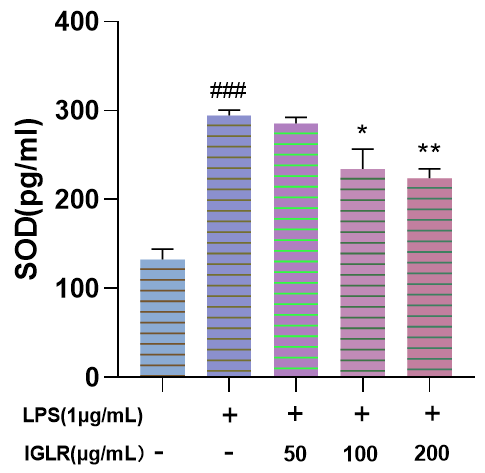 | 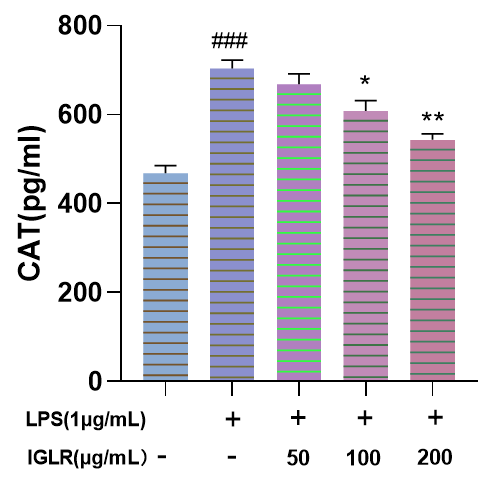 | 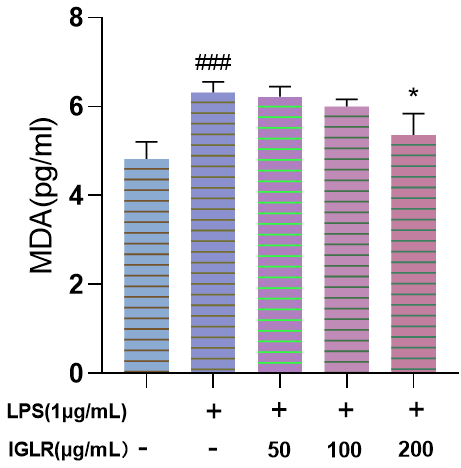 |

**Figure S2.** IGLR-CM scavenges oxygen free radicals. Effects of IGLR on SOD (**A**), CAT(**B**), and MDA (**C**) levels in L929 cells induced by IGLR-CM (0, 50, 100 and 200 μg/mL) for 24h (n = 3), ^###^*P* <0.005 compared with positive control group, **P* < 0.05, ***P* < 0.01 compared with LPS (1 μg/mL) group, one-way ANOVA with Tukey’s multiple comparison test. All data are reported as the means ± SEM.

| **A** | **B** | **C** | **D** |
| --- | --- | --- | --- |
| 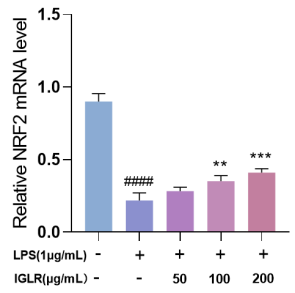 | 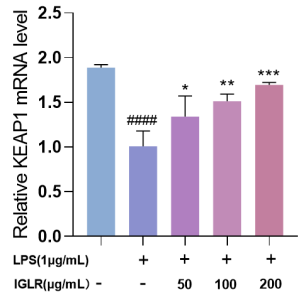 | 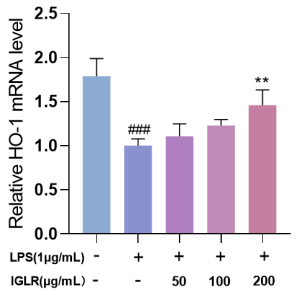 | 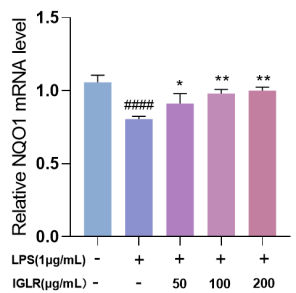 |

**Figure S3.** IGLR-CM remarkably elevated the level of antioxidative stress factors in fibroblasts. The mRNA level of NRF2 (**A**), KEAP1(**B**), HO-1(**C**) and NQO1 (**D**) with IGLR-CM by real-time PCR in L929 cells (n = 5), *^###^P* <0.005, *^####^P* < 0.001 compared with vehicle group, **P* < 0.05, ***P* < 0.01, ****P* < 0.005compared with LPS (1μg/mL) group, one-way ANOVA with Tukey’s multiple comparison test. All data are reported as the means ± SEM.

| **A** | **B** |
| --- | --- |
| 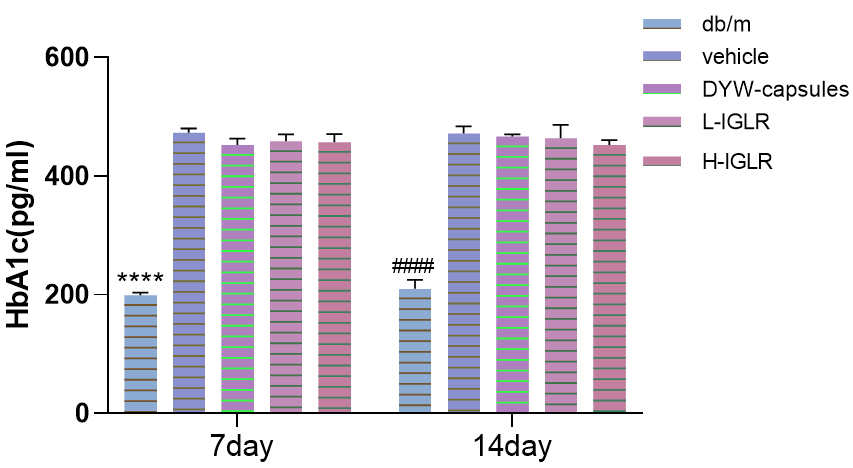 | 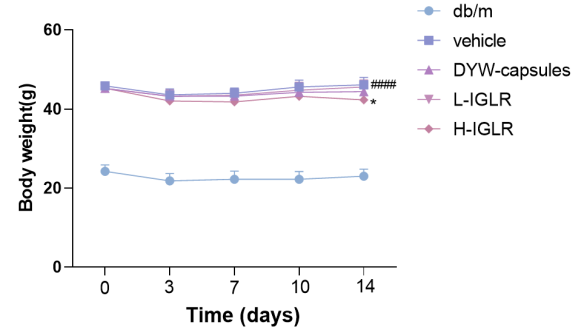 |

**Figure S4.** IGLR not significantly altered body weight and HbA1c levels in *db/db* mice. On days 0, 3, 7, 10 and 14 after wounding with various concentrations of IGLR to the body weight (**A**) and HbA1c level (**B**) in *db/db* mice (n = 6). *^####^P* < 0.001 compared with positive control group, **P* < 0.05 compared with vehicle group, one-way ANOVA with Tukey’s multiple comparison test. All data are reported as the means ± SEM.
